# Applicability of Hyaluronic Acid-Alginate Hydrogel and Ovarian Cells for *In Vitro* Development of Mouse Preantral Follicles

**DOI:** 10.22074/cellj.2020.6925

**Published:** 2020-09-08

**Authors:** Parisa Jamalzaei, Mojtaba Rezazadeh Valojerdi, Leila Montazeri, Hossein Baharvand

**Affiliations:** 1. Department of Anatomy, Faculty of Medical Sciences, Tarbiat Modares University, Tehran, Iran; 2. Department of Embryology, Reproductive Biomedicine Research Center, Royan Institute for Reproductive Biomedicine, ACECR, Tehran, Iran; 3. Department of Cell Engineering, Cell Science Research Center, Royan Institute for Stem Cell Biology and Technology, ACECR, Tehran, Iran; 4. Department of Developmental Biology, University of Science and Culture, Tehran, Iran; 5. Department of Stem Cells and Developmental Biology, Cell Science Research Center, Royan Institute for Stem Cell Biology and Technology, ACECR, Tehran, Iran

**Keywords:** Alginate, Fibrin, Hyaluronic Acid, Ovarian Cells, Preantral Follicle

## Abstract

**Objective:**

In the present study, the applicability of hyaluronic acid-alginate (HAA) hydrogel and ovarian cells (OCs) for the culture of mouse ovarian follicles were investigated and compared with those of alginate (ALG) and fibrin-alginate (FA) hydrogels.

**Materials and Methods:**

In the first step of this experimental study, mechanically isolated preantral follicles from the ovaries of two-week-old mice were encapsulated in the absence or presence of OCs in ALG, HAA, and FA hydrogels and cultured for 14 days. The morphology, diameter, survival and antrum formation rates of the follicles and the maturation and quality of the oocytes were evaluated during culture. In the second step, preantral follicles were cultured similar to the first step, but for 13 days, and their gene expressions and hormonal secretion were assessed on the last day of culture.

**Results:**

In the absence of OCs, higher numbers of ALG- and HAA-encapsulated follicles reached the antral stage compared to FA-encapsulated follicles (P<0.05). However, a higher percentage of HAA-developed oocytes resumed meiosis up to the germinal vesicle breakdown (GVBD)/metaphase II (MII) stages in comparison with ALG-developed oocytes (P<0.05). HAA-encapsulated follicles had significant overexpression of most of the growth and differentiation genes, and secreted higher levels of estradiol (E2) compared to ALG- and FA-encapsulated follicles (P<0.05). The co-culture condition increased the diameter of ALG-encapsulated follicles on day 13 of culture (P<0.05). It also increased the survival and maturation rates of ALG- and FA-encapsulated follicles, respectively (P<0.05). The co-culture condition improved cortical granule distribution in all groups, increased E2 and progesterone (P4) secretions in the ALG and FA groups, and androstenedione (A4) secretion in the FA group (P<0.05).

**Conclusion:**

The present study results show that HAA hydrogel is a promising hydrogel for follicle culture. OCs utilization could ameliorate the culture conditions regardless of the type of hydrogel.

## Introduction

Today, *in vitro* culture of isolated immature ovarian follicles would be a potential alternative for fertility preservation in adult or prepubertal patients with cancers that can metastasize to the ovaries (1, 2). In this regard, two systems namely, two-dimensional (2D, attachment) and three-dimensional (3D, non-attachment) have been developed. These systems support the growth of immature follicles *in vitro* to produce oocytes that can mature, become fertilized and result in live births in mice (3, 4). Unlike the 3D system, in the 2D system, follicle integrity is not preserved because of the poor correlation between the cultured follicle microenvironment and *in vivo* conditions (5). Therefore, in recent years, the 3D system has attracted more attention for follicle culture compared to the 2D technique and it has been proven that this system is more successful when translating to larger species (6).

One of the challenges that face the follicle culture in a 3D system is how to mimic the properties of the follicles in a physiological environment. Thus, it is important to apply appropriate biomaterials for follicle encapsulation (7). There are many potential natural and synthetic hydrogels, including agarose, hyaluronic acid (HA), collagen, fibrin, Matrigel, alginate (ALG), poly ethylene glycol (PEG) and their derivatives, which have been used for follicle culture (6, 8-11). ALG, a naturally-derived polysaccharide hydrogel produced from brown algae, has many of the characteristics that an optimal hydrogel requires for follicle culture (6). Follicles cultured in ALG hydrogel can grow and secrete hormones properly (6, 12). Despite documented positive outcomes, there are disadvantages associated with utilization of ALG hydrogel. The ALG degradation rate is not consistent with follicle growth rate, and this may affect oocyte maturation and increase abnormalities in cortical granule distribution, spindle formation, and chromosomal alignment (8, 13). Thus, oocytes that are cultured in ALG hydrogel have quite limited chances of becoming fertilized and reaching the blastocyst stage (12). In addition, ALG does not interact with the cells’ integrins, and this property affects the survival and proliferation of the follicular cells (14). To overcome these limitations, ALG can be combined with degradable compounds to make more appropriate hydrogels for the culture of the follicles without affecting its ideal properties. For example, Shikanov et al. added fibrin, as a degradable part to ALG (resulting in fibrinalginate hydrogel [FA]), and produced oocytes with higher quality and maturation rate (8). Fibrin is a protein derived from fibrinogen that is involved in blood clotting. It facilitates cell adhesion and is degraded by substances secreted by growing follicles. Therefore, fibrin provides dynamic cell-responsive biological and mechanical properties (15).

HA is an anionic glycosaminoglycan that can be combined with ALG to make a composite hydrogel suitable for follicle culture. HA is a primary component of the extracellular matrix (ECM) and is present in many organs, including the ovaries (16). The favourable properties of HA, such as its important role in cell migration, proliferation, and morphogenesis, enable it to make a physiologic milieu for follicle growth and development (9, 17, 18). Although it was shown that preantral follicles encapsulated in a HA hydrogel were able to resume meiosis, they lose out to increase the survival and antrum formation rates in comparison with ALG (18). These results might be attributed to the poor mechanical properties or lack of pores in the HA microstructure, which are necessary for follicle nutrition and growth (19). One way to optimize the poor mechanical properties of HA and form a porous microstructure is the creation of a composite hydrogel that consists of HA and ALG [hyaluronic acid-alginate hydrogel (HAA)]. Interestingly, the combination of ALG and HA in an HAA hydrogel can enhance both bioactivity and biodegradability of the ALG (20).

Although in many studies, ALG and FA have been used for encapsulation and culture of preantral follicles, the HAA hydrogel has not been used for this purpose. Importantly, no study has compared the effectiveness of these three types of hydrogels to introduce the most appropriate one for mouse ovarian follicle culture. Experimental evidence has emphasized the importance of molecular support for the ovarian environment, as well as its physical properties for follicular development (21). In this regard, recent studies have reported a stimulatory effect for ovarian cells (OCs) on the growth and differentiation of the follicles *in vitro* (22, 23). Hence, in the present study, the growth, survival, maturation, oocyte quality, gene expressions and hormonal secretions of the preantral follicles encapsulated in an HAA hydrogel in the absence or presence of OCs (OCs and +OCs-respectively) were evaluated in two steps. The results were compared with ALG- and FA-encapsulated follicles, which served as the control groups.

## Materials and Methods

### Study design

In the first step of the present experimental study, preantral follicles were isolated from mice ovaries in five independent replicates; randomly assigned to encapsulate in ALG, HAA and FA hydrogels in the absence or presence of OCs; and cultured for 14 days. The diameter and morphological appearance of the growing follicles were measured on days 1, 6, and 13 of culture. Moreover, on day 13 of culture, the survival rate of the follicles was evaluated, and the surviving follicles were assessed for antrum formation rate. After inducing the antral follicles with 2.25 IU/ml human chorionic gonadotropin (hCG, Choriomon, Switzerland) on day 13 of culture, the developmental stages of the oocytes obtained from induced antral follicles were determined on day 14 of culture. Finally, the qualities of MII oocytes in terms of cortical granule distribution, spindle formation, and chromosomal alignment were assessed. In the second step, follicles were isolated and cultured similar to the first step, but without hCG induction and in three independent replicates. On day 13 of culture, follicles were collected to evaluate their gene expressions, and the conditioned media were also gathered to measure follicle hormonal secretions. The study design has been summarized in Figure S1 (See Supplementary Online Information at www.celljournal.org).

### Animals

Female NMRI mice (Pasteur Institute of Iran) were kept in the animal house at Royan Institute with an adjusted temperature (20-25˚C) and lighting (12 hours light: 12 hours dark). They were handled according to the ethical guidelines set by Royan Institute (number: IR.ACECR. ROYAN.REC.1395.93).

### Ovarian cell isolation and culture

To obtain OCs (including theca/stromal cells, immune cells, endothelial cells, and smooth muscle cells), 20 mice that were three to four-weeks-old were sacrificed by cervical dislocation, and their ovaries were removed by an aseptic technique. Isolated ovaries were placed in ice-cold base medium that contained Dulbecco’s Modified Eagle’s medium (DMEM, Gibco, UK), streptomycin sulphate (Gibco, UK), penicillin (Gibco, UK), sodium bicarbonate (NaHCO_3_, Sigma, USA), and 10% foetal bovine serum (FBS, Gibco, UK). Next, the bursa and adipose tissues were removed under a stereomicroscope (SZ61, Olympus, Japan). Oocytes and granulosa cells were isolated from the ovaries by puncturing follicles using two 29G insulin syringes and then discarded. The remaining husks were minced and incubated for 45 minutes at 37˚C in 200 μl per ovary of collagenase solution that contained 4 mg/ ml collagenase IV (Gibco, UK) in a serum-free base medium. During this time, the ovarian tissues were pipetted at least 20 times every 10-15 minutes. The enzyme was inactivated by adding the same volume of the base medium. The digested cell solution was then filtered through a sterilized 40 μm filter mesh (Falcon, Mexico) and centrifuged at 1800 rpm for 5 minutes. The obtained cells were washed three times and the final pellet was resuspended in a certain volume of base medium. The cells were transferred to T25 culture flasks that contained 4 ml of base medium supplemented with 1% insulintransferrin- selenium (ITS, Gibco, UK), 1% non-essential amino acids (Gibco, UK), 1% L-glutamine (Sigma, USA), and 0.1% β-mercaptoethanol (Sigma, USA), and then cultured at 37˚C in a water-saturated atmosphere of 95% air and 5% CO_2_ until confluent. Then, the OCs were trypsinized, washed, and after centrifugation at 1800 rpm for 5 minutes, they were counted using a Neubauer chamber, and the cell survival was defined by Trypan blue staining (24). Finally, the cells were pelleted again in a 14 ml conical tube and 5×10^5^ cells were concentrated in 900 μl FBS. Then, 100 μl of dimethyl sulfoxide (DMSO, Sigma, USA) was added to the cell suspension. Afterward, 1 ml aliquots of the 10% DMSO solution were added to 1 ml cryovials and frozen at -80˚C until use.

### Follicle isolation

A total number of 60 mice (two-weeks-old) were sacrificed by cervical dislocation. Mouse ovaries were dissected under a stereomicroscope at 37˚C using two 29G insulin syringes in alpha minimum essential medium (α-MEM, Gibco, UK) supplemented with penicillin, streptomycin, NaHCO_3_, and 10% FBS. Only healthy preantral follicles that were 100-130 μm in diameter and two-three layers of granulosa cells were selected and divided randomly into the experimental groups.

### Hydrogel preparation

Alginic acid sodium salt (10 mg/ml, Sigma, USA), 4-(2-hydroxyethyl)-1-piperazineethanesulfonic acid (HEPES, 25 mM, Sigma, USA), and sodium chloride (NaCl, 150 mM, Sigma, USA) were dissolved in deionized water to make a 1.0% (w/v) ALG solution (20). Immediately before use, the sterilized ALG solution was reconstituted with sterile 1X phosphate-buffered saline (PBS, Takara, Japan) without calcium and magnesium to yield a 0.5% (w/v) concentration. FA solution was prepared by mixing fibrinogen solution [50 mg/ml fibrinogen (Sigma, USA) in 3000 KIU/mL aprotinin (Roche, Germany)] with 1% ALG solution at 1:1 ratio. HAA solution was made by the addition of HA [5 mg/ml, Nano Zist Arrayeh (NZA), Iran] to a 0.5% ALG solution (8, 20).

In order to prepare the hydrogels, cross-linking solutions [50 mM calcium chloride (CaCl_2_, Sigma, USA)/140 mM NaCl for making ALG and HAA, and 50 mM CaCl_2_/140 mM NaCl with the equal volume of 50 IU/ml thrombin solution for FA] were mixed with the hydrogel solutions.

### Encapsulation and culture

In the first step of the study, groups of 94.66 ± 1.54 preantral follicles were individually encapsulated in ALG, HAA, and FA solutions and in the absence or presence of OCs, in five independent replicates. A schematic representation of the co-encapsulation of follicles and OCs is depicted in Figure S2 (See Supplementary Online Information at www.celljournal.org). In detail, for cell encapsulation, thawed and cultured OCs were washed twice in PBS, detached using trypsin-EDTA, and counted. After pelleting, a certain number of cells (5×10^3^ cells per follicle based on the best results obtained in the pilot stage of the study) was mixed with hydrogel solutions and pipetted in 5 μl droplets on sterile ultra-low attachment culture dishes (Dow Corning, USA). Follicles were individually cultured in microdrops (5 μl) and crosslinking solutions were gently pipetted on top of each droplet, and then incubated at 37˚C for 2 and 5 minutes. After incubation, ALG, HAA, and FA beads were rinsed with medium and then placed into 96-well plates (TPP, Switzerland). Each well contained one bead in 100 μl culture medium [α-MEM supplemented with 5% FBS, 1% ITS, 10 mIU/ml follicle stimulating hormone (FSH, Merck, Germany)]. Finally, the plates were incubated in 5% CO_2_ at 37˚C for 13 days and 50 μl of the medium was replaced every 3 days.

### Evaluation of follicle diameters, survival, and antrum formation rates

On days 1, 6, and 13 of culture, morphological features of the follicles were assessed, and the diameters of the growing follicles were defined as the average of two perpendicular diameters of every follicle using ImageJ software (U.S. National Institutes of Health). On day 13 of culture, the survival rate of the cultured follicles and antrum formation rate of the surviving follicles were observationally evaluated. For assessment of follicle survival rate, extrusion of the oocytes and a dark appearance of the oocytes and surrounding granulosa cells were considered to be signs of degeneration. In addition, antrum formation was defined as a noticeable lucid cavity within masses of granulosa cells.

### Determination of oocyte maturation

On day 13 of culture, *in vitro* maturation (IVM) and ovulation of antral follicles were induced by 2.25 IU/ ml hCG. To determine oocyte maturation, 20–22 hours after stimulation, cumulus-oocyte complexes (COCs) were isolated from induced follicles by their suction into Pasteur pipettes, their cumulus cells were eliminated by gentle pipetting, and the number of germinal vesicle (GV), germinal vesicle breakdown/MII (GVBD/MII), and degenerated oocytes were calculated.

### Assessment of cortical granule distribution, spindle formation, and chromosomal alignment

A total number of 15 *in vivo*- and 90 *in vitro*-developed MII oocytes (15 oocytes per group) were randomly collected in three independent replicates for this assessment. To obtain *in vivo*-developed oocytes as the control group, three female 6–8 week-old mice were injected intraperitoneally with 7.5 IU of pregnant mare serum gonadotropin (PMSG, Sigma, USA) followed by administration of 7.5 IU hCG 48 hours later. After 18 hours, the mice were sacrificed, their COCs were removed from the oviduct ampulla and denuded by gentle pipetting in 0.3% hyaluronidase solution (Sigma, USA). Then, the zona pellucida of *in vivo*-developed MII oocytes along with *in vitro*-developed ones were removed by 0.5 mg/ ml pronase (Sigma, USA) in PBS at 37˚C and then the oocytes were fixed in 4% paraformaldehyde for at least 1 hour. After washing in PBS with 0.01% Tween 20 (Sigma, USA), the oocytes were permeabilized in PBS with 0.3% bovine serum albumin (BSA, Gibco, UK) and 0.1% Triton X-100 (Sigma, USA) for 15 minutes, and then blocked in PBS that contained 0.3% BSA. In order to visualize the meiosis spindle and cortical granules, the oocytes were incubated with a mixture of anti-alpha-tubulin antibody-microtubule marker (FITC, 1:100; Abcam, UK) and rhodamine-labeled Lens Culinaris Agglutinin (LCA, 1:500, Vector Laboratories, USA) in blocking solution for 1 hour at 37˚C. Finally, the stained oocytes were washed thoroughly in PBS-T, counterstained with Hoechst 33342 (1 mg/ml in 1X PBS, Sigma, USA) for 5 minutes at 37˚C and mounted on adhesion slides (13). Fluorescence was detected using an inverted fluorescence microscope (Eclipse 50i, Nikon, Japan) and images were processed using Adobe Photoshop software (CS5.1, Adobe Systems, Inc., San Jose, CA, USA). The absence of a cortical granule-free domain (CGFD) around the spindle and lack of a cortical distribution, and disorganized spindle configuration or misaligned chromosomes were respectively considered as the signs of cortical granule and spindle abnormalities.

### Evaluation of gene expression

In the second step, to evaluate gene expressions, a total number of 75 antral follicles (25 follicles per replicate) that survived were pooled in each group, in three replicates, on day 13 of culture. The follicles were retrieved from the hydrogel beads by gentle suction into Pasteur pipettes. Total RNA was extracted using the RNeasy Micro Kit (Qiagen, Germany) according to the manufacturer’s protocol. Synthesis of cDNA was performed using a RevertAid first-strand cDNA synthesis kit (Fermentas, Germany) and random hexamer based on the manufacturer’s instructions. The expressions of seven growth and differentiation genes, five apoptotic genes, and five genes involved in steroidogenesis were assessed using RT-qPCR. Primers for the mentioned and housekeeping genes (GAPDH) were designed using Allele ID (v.6, Premier Biosoft, USA) and PerlPrimer (v.1.1.21, http://perlprimer.sourceforge.net/) primer design softwares (Table S1, See Supplementary Online Information at www.celljournal.org). The polymerase chain reaction (PCR) mix for each well contained 5 ml Power SYBR Green PCR Master Mix (Takara, Japan), 1 ml dH_2_O, 1 ml of each of the forward and reverse primers (5 pmol/ml), and 2 ml of single-strand cDNA in a final reaction volume of 10 ml. PCR was performed on the ABI StepOnePlus real-time PCR system (Applied Biosystems) using the following program: stage 1: 95˚C for 10 minutes; stage 2: 40 cycles of 95˚C for 15 seconds and 60˚C for 1 minute; and stage 3: 95˚C for 15 seconds, 60˚C for 15 seconds, and 95˚C for 15 seconds. Product specificity was confirmed by melting curve analysis. All samples were assessed in duplicate, and for each reaction, a no-template control reaction (NTC) was run. The expression of genes was compared between groups using the ∆CT method. Gene expression levels were normalized against *GAPDH*.

### Measurement of hormonal secretions

Following evaluation of gene expression, the levels of estradiol (E2), progesterone (P4), and androstenedione (A4) were also measured in conditioned media collected from 30 cultured antral follicles per group (10 follicles per replicate) on day 13 of culture. Hormonal secretions were assessed by mouse ELISA kits (Bioassay Technology Laboratory, China) according to the kits’ instructions. Data were calculated for each follicle by dividing each of the measured hormone secretions by the number of the follicles. According to the kits’ datasheets, the sensitivity assays for E2, P4 and A4 were 1.51, 0.28 and 0.022 ng/ ml, respectively.

### Statistical analysis

Binary data, including the proportion of the follicle survival and antrum formation rates, oocyte maturation and abnormality rates were analysed using the GENMOD procedure including the logit link function model. The GENMOD procedure produced the odds ratio (OR) as the strength of the difference between groups. Data associated with follicle diameter over the course of the culture were analysed by the MIXED procedure including RANDOM and REPEATED statements in the model to specify between and within covariances, respectively. Data pertaining to gene expression and hormonal secretion were analysed using the GLM procedure. In HAA Hydrogel and Follicle Development 53 Cell J, Vol 22, Suppl 1, Autumn 2020 addition, the LSMEANS statement was included in the model to perform multiple comparisons. All analyses were conducted in SAS version 9.4 (SAS Institute Inc., NC, USA). Differences were considered significant at P<0.05.

## Results

### Follicle morphological characteristic and diameter

**Fig.1:**
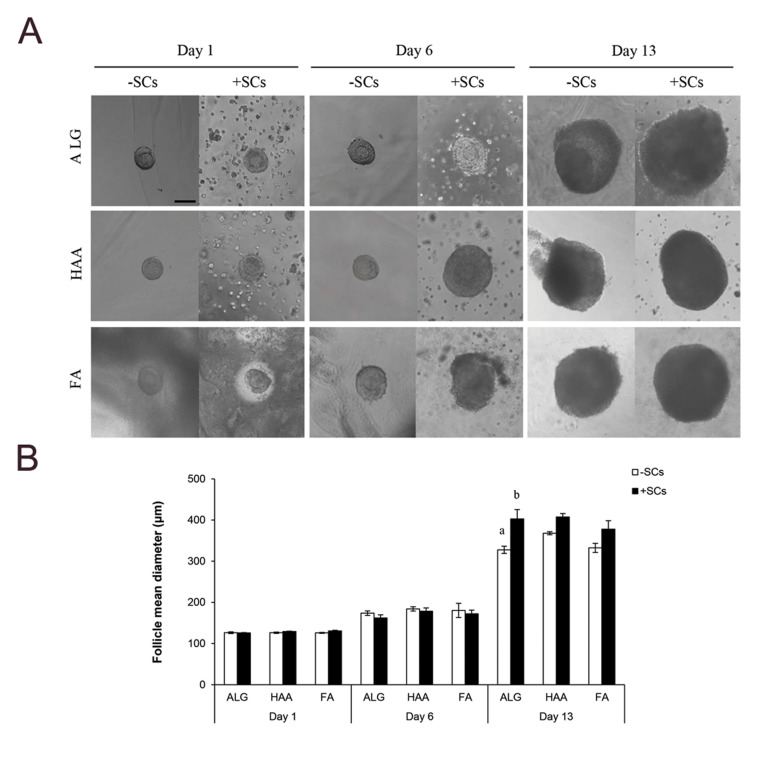
Growth of preantral follicles encapsulated and cultured in alginate (ALG), hyaluronic acid-alginate (HAA) and fibrin-alginate (FA) hydrogels in the absence or presence of ovarian cells (OCs and +OCs-respectively). **A.** Morphological changes and **B.** Diameters of the surviving follicles on days 1, 6, and 13 of culture. Data are presented as mean diameter ± standard error (SE). Data points a and b differ significantly (P<0.05, scale bar: 100 μm). OCs; ovarian Cells.

Assessment of morphological changes and follicle diameter after 1, 6, and 13 days of culture showed that there was no significant difference in the morphology and diameter of the follicles encapsulated in different hydrogels, either in the absence or presence of OCs. Nevertheless, the follicles co-cultured with OCs had a more spherical shape and a larger diameter. On day 13 of culture, the difference in diameter of -OCs and +OCs follicles was significant in ALG encapsulated ones (327.59 ± 8.74 vs. 402.73 ± 22.63 μm, respectively; P<0.05, Fig .1A, B).

### Survival rate and antrum formation

Evaluation of survival rate after 13 days of culture showed that in the absence of OCs, the survival rate of ALG-encapsulated follicles (69.47%) was significantly higher than FA-encapsulated (53.06%, P<0.05, Table 1). However, in the presence of OCs, the survival rate of ALG-, HAA- and FA-encapsulated follicles did not differ. On the other hand, the addition of OCs to FA hydrogel beads significantly increased the number of surviving follicles (53.06 vs. 75% for FA-OCs and FA+OCs, P<0.05, Table 1).

Antrum formation results revealed that in the absence of OCs, higher numbers of ALG- (81.81%) and HAA- (82.25%) encapsulated follicles reached the antral stage compared to FA-encapsulated follicles (69.23%, P<0.05, Table 1). Nonetheless, in the presence of OCs, the antrum formation did not vary significantly between the ALG, HAA, and FA groups. Also, a comparison of the -OCs and +OCs groups showed that the co-culture of follicles with OCs did not influence follicle antrum formation.

### Oocyte maturation

Table 1 shows various developmental stages of oocytes obtained from follicles cultured in ALG, HAA, and FA hydrogels, in the absence or presence of OCs. Data revealed that in the absence of OCs, a higher percentage of HAA-developed oocytes resumed meiosis up to the GVBD/MII stages in comparison with ALG-developed oocytes (74.50 vs. 55.55%, P<0.05), while in the presence of OCs there was no significant difference between groups. Assessment of -OCs and +OCs groups also confirmed that the oocytes which were co-cultured with OCs were more likely to break down their GVs and reach the GVBD/MII stages, whereas in the ALG group this difference was remarkable (GV% oocytes: 29.62 vs. 10.14%, GVBD /MII% oocytes: 55.55 vs. 72.46% for-OCs and +OCs, respectively, P<0.05).

### Cortical granule distribution, spindle formation, and chromosomal alignment

**Fig.2:**
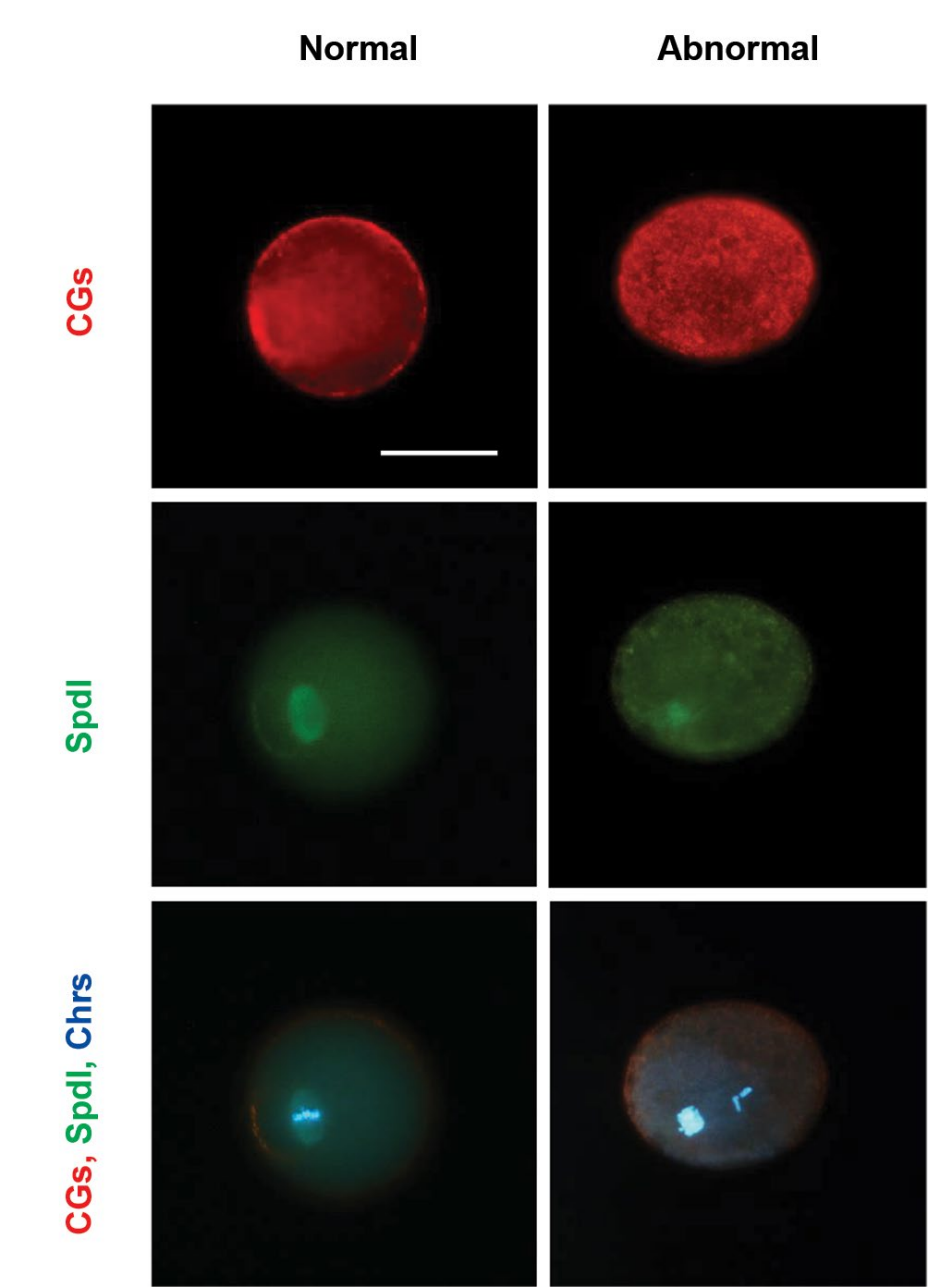
Immunofluorescence staining of cortical granules (CGs, red), meiotic spindle (Spdl, green) and chromosomes (Chrs, blue) in normal and abnormal metaphase II (MII) oocytes. The absence of a cortical granulefree domain (CGFD) around the spindle and lack of a cortical distribution, and disorganized spindle configuration or misaligned chromosomes were respectively considered the signs of cortical granules and spindle abnormalities (scale bar: 50 μm).

Figure 2 shows the features of the normal and abnormal MII oocytes in terms of cortical granule distribution, meiotic spindle organization, and chromosomal alignment.

Data indicated that none of the *in vivo* developed oocytes showed any abnormalities in cortical granule distribution, whereas it appeared to be impaired in the oocytes from all of the *in vitro* groups (P<0.05). Evaluation of *in vitro* developed oocytes revealed that there was no significant difference between the ALG, HAA, and FA groups neither in the absence nor in the presence of OCs. However, a comparison of the -OCs and +OCs groups showed that co-culturing with OCs improved the abnormalities in the distribution of the cortical granules (P<0.05, Table 2).

In terms of meiotic spindle and chromosomal alignment, abnormalities were observed in both *in vivo* and *in vitro* developed oocytes. Importantly, neither the type of hydrogel nor the presence of OCs had significant effects on the rate of abnormalities (Table 2).

### Gene expression

In the second step of the study, the expressions of some of the genes involved in growth and differentiation of follicles (*Gdf9, Bmp15, Zp3, Gja4, Gja1, Bmp4, *and *Bmp7*), apoptosis (*Trp53, Casp3, Bax, *and *Bcl2*) and steroidogenesis (*Fshr, Lhcgr, Cyp11a1, Cyp17a1, *and *Cyp19a1*) were studied (Fig . 3).

**Table 1:** Development of preantral follicles cultured in ALG, HAA and FA hydrogels in the absence or presence of OCs for 14 days


Groups	Survival rate		Antrum formation rate		Oocyte maturation					
					GV		GVBD/MII		Degenerated	
	-OCs	+OCs	-OCs	+OCs	-OCs	+OCs	-OCs	+OCs	-OCs	+OCs

ALG	66/95 (69.47)^A^	78/98 (79.59)	54/66 (81.81)^A^	69/78 (88.46)	16/54 (29.62)^a^	7/69 (10.14)^b^	30/54 (55.55)^Aa^	50/69 (72.46)^b^	8/54 (14.81)	12/69 (17.39)
HAA	62/93 (66.66)	71/96 (73.95)	51/62 (82.25)^A^	63/71 (88.73)	7/51 (13.72)	6/63 (9.52)	38/51 (74.50)^B^	53/63 (84.12)	6/51 (11.76)	4/63 (6.34)
FA	52/98 (53.06)^Ba^	66/88 (75)^b^	36/52 (69.23)^B^	50/66 (75.75)	10/36 (27.77)	7/50 (14)	23/36 (63.88)	38/50 (76)	3/36 (8.33)	5/50 (10)


Data are presented as n (%). Data points A and B in each column, a and b in each row differ significantly (P<0.05). ALG; Alginate, HAA; Hyaluronic acidalginate, FA; Fibrin-alginate, -OCs; Culture in the absence of stromal cells, +OCs; Culture in the presence of stromal cells, GV; Germinal vesicle, GVBD; Germinal vesicle breakdown, and MII; Metaphase II.

**Table 2:** Abnormality rate of *in vivo* and *in vitro* developed MII oocytes on day 14 of culture


Groups	In vivo	ALG		HAA		FA	
		-OCs	+OCs	-OCs	+OCs	-OCs	+OCs

Abnormal CGs	0/15 (0)^a^	10/15 (66.66)^ bc^	6/15 (40)^ bd^	15/15 (100)^be^	9/15 (60)^bf^	15/15(100)^bg^	8/15 (53.33^)bh^
Abnormal Spdl and Chrs	3/15 (20)	7/15 (46.66)	7/15 (46.66)	4/15 (26.66)	7/15 (46.66)	3/15 (20)	4/15 (26.66)


Data are presented as n (%). Data points a and b, c and d, e and f, g and h differ significantly (P<0.05). MII; Metaphase II, ALG; Alginate, HAA; Hyaluronic acid-alginate, FA; Fibrin-alginate, -OCs; Culture in the absence of stromal cells, +OCs; Culture in the presence of stromal cells, CGs; Cortical granules, Spdl; Spindle, and Chrs; Chromosomes.

In general, in the absence of OCs, all growth and differentiation genes, except for *Gja1* and *Bmp4*, were significantly overexpressed in the HAA group (P<0.05), while the expressions of pro-apoptotic genes, *Trp53* and *Bax* in the ALG group were significantly higher than those of the FA group (P<0.05). *Bax* expression was also significantly higher in the HAA group (P<0.05). Moreover, the investigation of pro-apoptotic and antiapoptotic genes and *Bax/Bcl2* ratio revealed a higher tendency for apoptosis in the follicular cells of the ALG group in comparison to the HAA and FA groups. On the other hand, FA-encapsulated follicles expressed *Lhcgr, Cyp17a1*, and Cyp19a, as steroidogenic genes, at much higher levels compared to HAA-encapsulated and ALG-encapsulated follicles (P<0.05); however, the expressions of *Fshr* and *Cyp11a1* genes did not differ significantly between groups.

In the presence of OCs, only the expression level of *Bmp7* was higher in HAA-encapsulated follicles compared to ALG- encapsulated and FA-encapsulated follicles (P<0.05). No significant difference in the expressions of the apoptotic genes was observed between groups; nonetheless, like -OCs, FAencapsulated follicles expressed *Cyp19a1* at much higher levels compared to the ALG-encapsulated and HAA-encapsulated follicles (P<0.05).

Finally, comparable results between the -OCs and +OCs groups indicated that adding OCs to the hydrogel beads did not have any significant effect on expressions of the growth and differentiation genes, but it did decrease the expressions of *Trp53* in the ALG group and *Bax* in the ALG and HAA groups (P<0.05), and decreased the expression of *Lhcgr* in the FA group (P<0.05).

**Fig.3:**
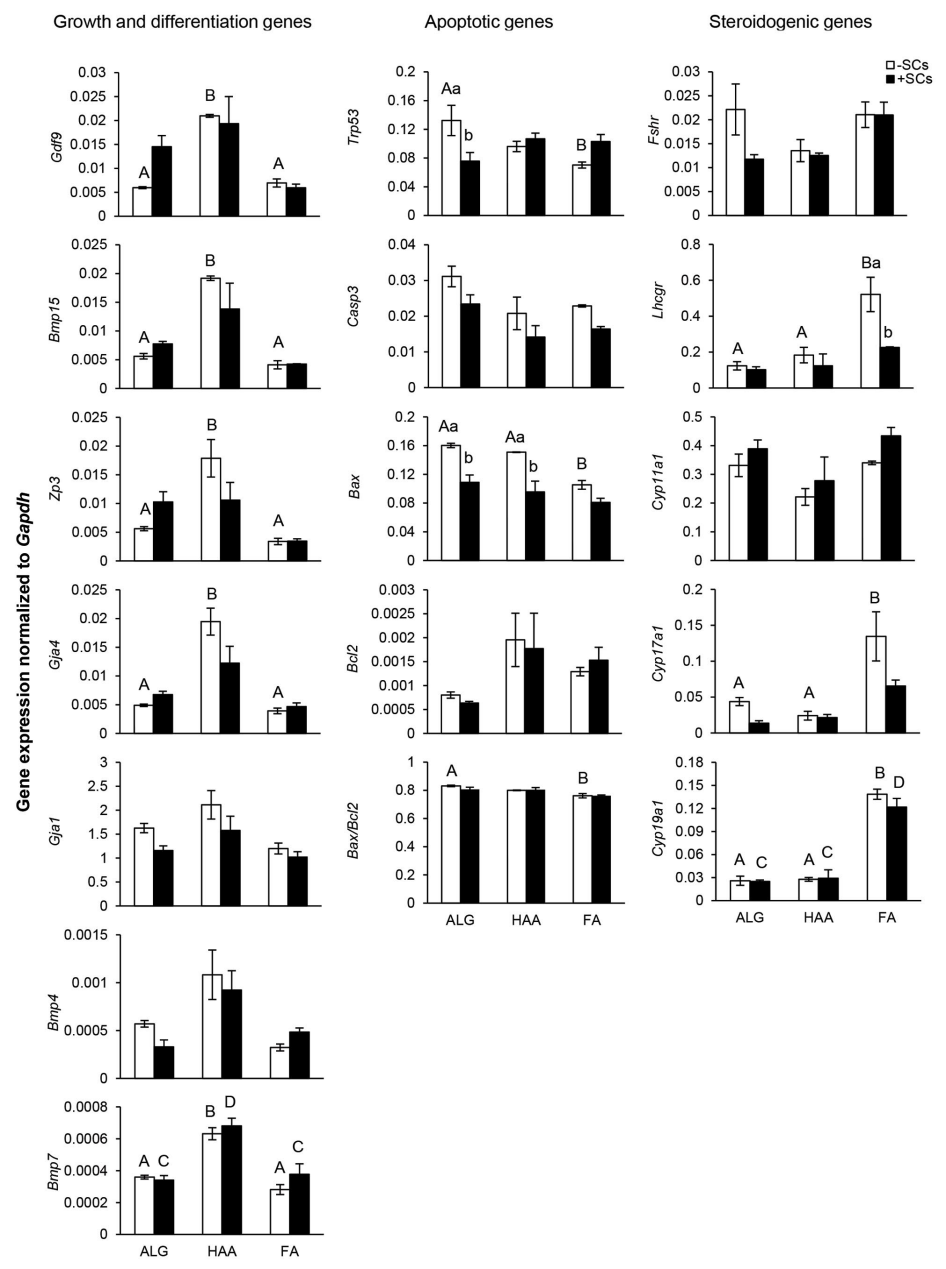
Expression of growth and differentiation (*Gdf9, Bmp15, Zp3, Gja4, Gja1, Bmp4,* and *Bmp7*), apoptotic (*Trp53, Casp3, Bax, *and *Bcl2*) and steroidogenic (*Fshr, Lhcgr, Cyp11a1, Cyp17a1,* and *Cyp19a1*) genes in follicles encapsulated and cultured in alginate (ALG), hyaluronic acid-alginate (HAA) and fibrinalginate (FA) hydrogels in the absence or presence of ovarian cells (OCs and +OCs-respectively). Antral follicles were collected on day 13 of culture. Expression levels were normalized to *GAPDH* as the endogenous control. Data are presented as mean ± standard error (SE). Data points A and B, C and D, a and b differ significantly (P<0.05).

### Hormonal secretion

**Fig.4:**
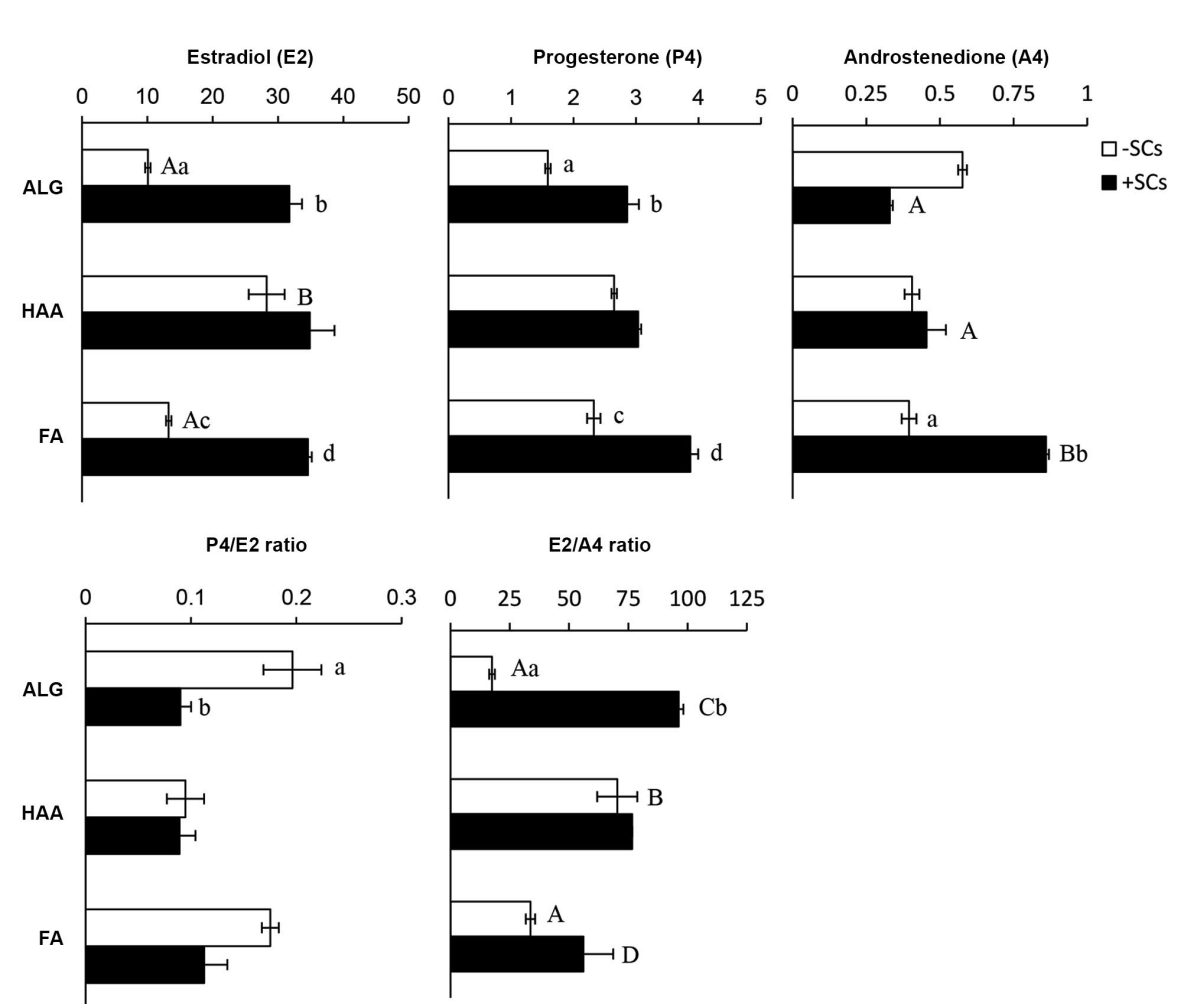
Secretion of estradiol (E2), progesterone (P4), and androstenedione (A4) by follicles encapsulated and cultured in alginate (ALG), hyaluronic acid-alginate (HAA) and fibrin-alginate (FA) hydrogels in the absence or presence of ovarian cells (OCs and +OCs-respectively). Conditioned media were collected on day 13 of culture. Data are presented as mean ± standard error (SE). Data points A and B, C and D, a and b differ significantly (P<0.05).

We assessed the E2, P4, and A4 secretions by the cultured follicles, and the P4/E2 and E2/A4 ratios (Fig .4). Data indicated that in the absence of OCs, HAA-encapsulated follicles (28.27 ± 1.67 ng/ml) secreted higher levels of E2 compared to ALG-encapsulated (10.08 ± 0.79 ng/ml) and FAencapsulated (13.26 ± 0.06 ng/ml, P<0.05) follicles; however, no significant difference was found between groups in the levels of P4 and A4. Furthermore, the P4/E2 ratio did not significantly vary between groups, but the E2/A4 ratio in the HAA group was higher than the other groups due to higher E2 levels in the HAA group (P<0.05). Nonetheless, in the presence of OCs, there was no significant difference in E2 and P4 secretions between groups, but the A4 level in FAencapsulated follicles was significantly higher than the ALG-encapsulated and HAA-encapsulated (0.86 ± 0.02 vs. 0.33 ± 0.01 and 0.45 ± 0.02 ng/ml, P<0.05) follicles. In the presence of OCs, the P4/E2 ratio did not vary significantly between groups, but the E2/A4 ratio in the ALG group was higher than the FA group (P<0.05). Finally, a comparison of the -OCs and +OCs groups showed that co-culture of the follicles with OCs increased E2 secretion in the ALG (10.08 ± 0.79 ng/ml) and FA groups (31.74 ± 0.30 ng/ml) for -OCs and in the ALG (13.26 ± 0.06 ng/ml) versus FA (34.57 ± 1.12 ng/ml) groups for +OCs (P<0.05). There was also increased P4 secretion in the ALG and FA groups (1.59 ± 0.03 vs. 2.86 ± 0.35 and 2.32 ± 0.09 vs. 3.87 ± 0.63 ng/ml for-OCs and +OCs, respectively, P<0.05). There was increased A4 secretion in the FA group (0.39 ± 0.02 vs. 0.86 ± 0.02 ng/ml for-OCs and +OCs, respectively, P<0.05). Additionally, the P4/E2 and E2/A4 ratios in ALG-OCs group were significantly higher and lower than that of the ALG+OCs group, respectively (P<0.05).

## Discussion

The aim of the present study was to investigate the applicability of HAA hydrogel and OCs for the culture of ovarian follicles. In this regard, the diameter, survival and antrum formation rates, gene expression profile, and steroidogenic activity of cultured follicles, and developmental competence and quality of obtained oocytes were evaluated.

In the absence of OCs, a comparison of the ALG, HAA, and FA groups showed that the antrum formation and GV to GVBD/MII transition rates in the HAA group were relatively higher than the ALG and FA groups. Based on the mentioned results, it could be proposed that HAA hydrogel supported the growth and development of follicles better than the other evaluated hydrogels.

Nevertheless, the majority of the cultured oocytes in -OCs groups (HAA, ALG, and FA) did not show a normal distribution of cortical granules (important granules within oocyte cytoplasm that are involved in polyspermypreventing mechanisms) (25). These observations were in line with Mainigi et al., who reported that oocytes obtained from follicles cultured in ALG hydrogel exhibited abnormalities in cortical granule distribution (13). The stated findings could be due to the clumping of cortical granules across the cytoplasm either as a result of increased Ca^2+^ concentration or altered expression of some proteins, which are involved in cortical granule fusion and exocytosis (13, 26). Therefore, it can be suggested that *in vitro* culture of follicles increases the oocyte susceptibility to errors in cortical granule distribution during maturation, regardless of the type of the matrix.

Based on our findings, in the absence of OCs, the HAA-encapsulated follicles highly expressed all growth and differentiation genes with the exception of *Gja1* and *Bmp4. Gdf9, BMP15*, and *BMP7* play important roles in follicular growth and development and cumulus expansion (27-29). Moreover, *Gja4* contributes to the gap junctions between the oocyte and the surrounding granulosa cells (30). On the other hand, *Zp3* encodes the most abundant protein in the oocyte’s zona pellucida (31). Therefore, overexpression of the mentioned genes in the HAAOCs group could represent effective oocyte-granulosa cell communication and more marked development of follicles, subsequently, high-quality fertilization and embryo development in this group.

Apoptosis or programmed cell death is modulated by several hormones and growth factors as well as intrinsic factors like TRP53, BAX, BCL2, and CASP3 (32, 33). In our study, in the absence of OCs, the *Bax/Bcl2* ratio, as well as Trp53 and Casp3 expressions in the HAA group, did not significantly differ from those of the ALG and FA groups. Hence, it could be deduced that apoptosis occurred in the HAA group at similar rates to the ALG and FA groups.

Nonetheless, it was shown that in the absence of OCs, ALG-encapsulated and HAA-encapsulated follicles expressed *Lhcgr, Cyp17a1* and *Cyp19a1* genes at a similar level but much lower than those of FA-encapsulated follicles. Gonadotropic hormones, FSH and LH, induce granulosa and theca/stromal cells via their receptors, FSHR and LHCGR, respectively. Afterwards, *CYP17A1* produces P4 and A4 from cholesterol, and *CYP19A1* produces E2 from A4 (34, 35). Accordingly, it is suggested that both the granulosa and theca cells in FA-encapsulated follicles are highly active in steroidogenesis. However, without evaluation of E2, P4, and A4 secretions, it is not possible to approve their health and correct functionality.

In the absence of OCs, FA follicles secreted both E2 and A4 at low levels as reflected by a low E2/A4 ratio. This observation was unexpected as they overexpressed *Cyp17a1* and *Cyp19a1* genes. Since the overexpression of *LHCGR, CYP17A1, *and *CYP19A1*, and the recorded pattern of steroidogenesis are regarded as negative indicators of follicle health, it could be suggested that the FA hydrogel did not have any favourable effects on follicle culture. Presumably, follicle functionality was affected through alteration of regulatory elements such as transcription factors or some intracellular signalling pathways that mediate the expression and translation of steroidogenic genes (36). Unlike ALG and FA, HAAencapsulated follicles secreted higher amounts of E2, which resulted in a higher E2/A4 ratio. This pattern of the hormonal secretions is acceptable for large antral follicles and may prove the health of the HAA group’s follicles and their proper functionality.

In the presence of OCs, there was no significant difference between encapsulated follicles in the ALG, HAA, and FA groups in terms of follicle diameter, survival, antrum formation and maturation rates, oocyte abnormality rate, gene expression (except for *Bmp7 *and *Cyp19a1*), E2 and P4 secretions, and the P4/E2 ratio. Therefore, it can be suggested that OCs may relatively change hydrogel bead properties due to the secretion of some enzymes such as protease, and make the culture condition identical to the follicles of all groups (8).

Finally, a comparison of the -OCs and +OCs groups indicated that the follicles in +OCs groups had a more regular shape, better growth and developmental conditions compared to -OCs groups (including a larger diameter, as well as higher survival and GV to GVBD/MII transition rates, but a lower oocyte abnormality rate). Previous studies have shown that OCs have a high secretion activity and can reproduce the theca cell layer when co-cultured with follicles, consequently enhancing the growth and development of follicles *in vitro* (22, 23). In our study, the expression of growth and differentiation genes, especially *Bmp4* and *Bmp7* that are highly expressed in theca/ stromal cells, was similar in -OCs and +OCs groups. So, it could be concluded that co-culturing OCs probably did not contribute to the formation of the theca cell layer, and exerted their effect on follicles by secreting growth and development factors that changed the expression of some other genes that we did not check. Furthermore, the coculture of the follicles with OCs decreased the expressions of *Trp53* and *Bax* in the ALG and HAA groups. However, it did not significantly affect *Casp3* expression and the *Bax/Bcl2* ratio. Thus, it can be suggested that in contrast to the findings of the previous studies, OCs could not suppress the apoptosis process within the follicular cells (37). The co-culture condition also did not change the expressions of steroidogenic genes, except for *Lhcgr*, in the FA group; nonetheless, it enhanced secretion of E2 in the ALG and FA groups, and A4 in the FA group. This inconsistency between gene expression and hormonal secretion might be explained as follows. The evaluation of gene expression was only performed on large antral follicles without surrounding co-cultured OCs, but hormone secretion was evaluated on conditioned media from both follicles and OCs. In this regard, although no significant difference in gene expression was observed between the -OCs and +OCs groups, hormonal secretion varied significantly between these groups.

## Conclusion

The present study showed that, in the absence of OCs, the applied HAA hydrogel caused promising effects on follicle culture. Indeed its suitable stiffness and desirable innate attributes improved growth and development of the follicles by affecting some biochemical pathways that remain to be discovered in future studies. Furthermore, co-culturing the follicles with OCs improved the culture condition regardless of the type of hydrogel they were encapsulated in. These cells could be used properly in follicle culture systems in order to ameliorate the culture condition.

## Supplementary PDF


