# Supplementary material for: Applicability of Hyaluronic Acid-Alginate Hydrogel and Ovarian Cells for In Vitro Development of Mouse Preantral Follicles
Source: Cell J. 2020 Sep 8;22(Suppl 1):49–60. doi: 10.22074/cellj.2020.6925 (PMC7481901; doi:10.22074/cellj.2020.6925)
Supplement: Supplementary file 1 [file Cell-J-22-Suppl1-49-s01.pdf]

Supplementary Information for

Applicability of Hyaluronic Acid-Alginate Hydrogel and Ovarian Cells for *In Vitro* Development of Mouse Preantral Follicles

Parisa Jamalzaei, Ph.D.[1], Mojtaba Rezazadeh Valojerdi, Ph.D.[1, 2*], Leila Montazeri, Ph.D.[3], Hossein Baharvand, Ph.D.[4, 5*]

1. Department of Anatomy, Faculty of Medical Sciences, Tarbiat Modares University, Tehran, Iran
2. Department of Embryology, Reproductive Biomedicine Research Center, Royan Institute for Reproductive Biomedicine, ACECR, Tehran, Iran
3. Department of Cell Engineering, Cell Science Research Center, Royan Institute for Stem Cell Biology and Technology, ACECR, Tehran, Iran
4. Department of Developmental Biology, University of Science and Culture, Tehran, Iran
5. Department of Stem Cells and Developmental Biology, Cell Science Research Center, Royan Institute for Stem Cell Biology and Technology, ACECR, Tehran, Iran

**Corresponding Address: P.O.Box: 14115-111, Department of Anatomy, Faculty of Medical Sciences, Tarbiat Modares University, Tehran, Iran*
P.O.Box: 16635-148, Department of Embryology, Reproductive Biomedicine Research Center, Royan Institute for Reproductive Biomedicine, ACECR, Tehran, Iran
Email: mr_valojerdi@modares.ac.ir
P.O. Box: 13145-871, Department of Developmental Biology, University of Science and Culture, Tehran, Iran
P.O.Box: 16635-148, Department of Stem Cells and Developmental Biology, Cell Science Research Center, Royan Institute for Stem Cell Biology and Technology, ACECR, Tehran, Iran
Email: baharvand@royaninstitute.org

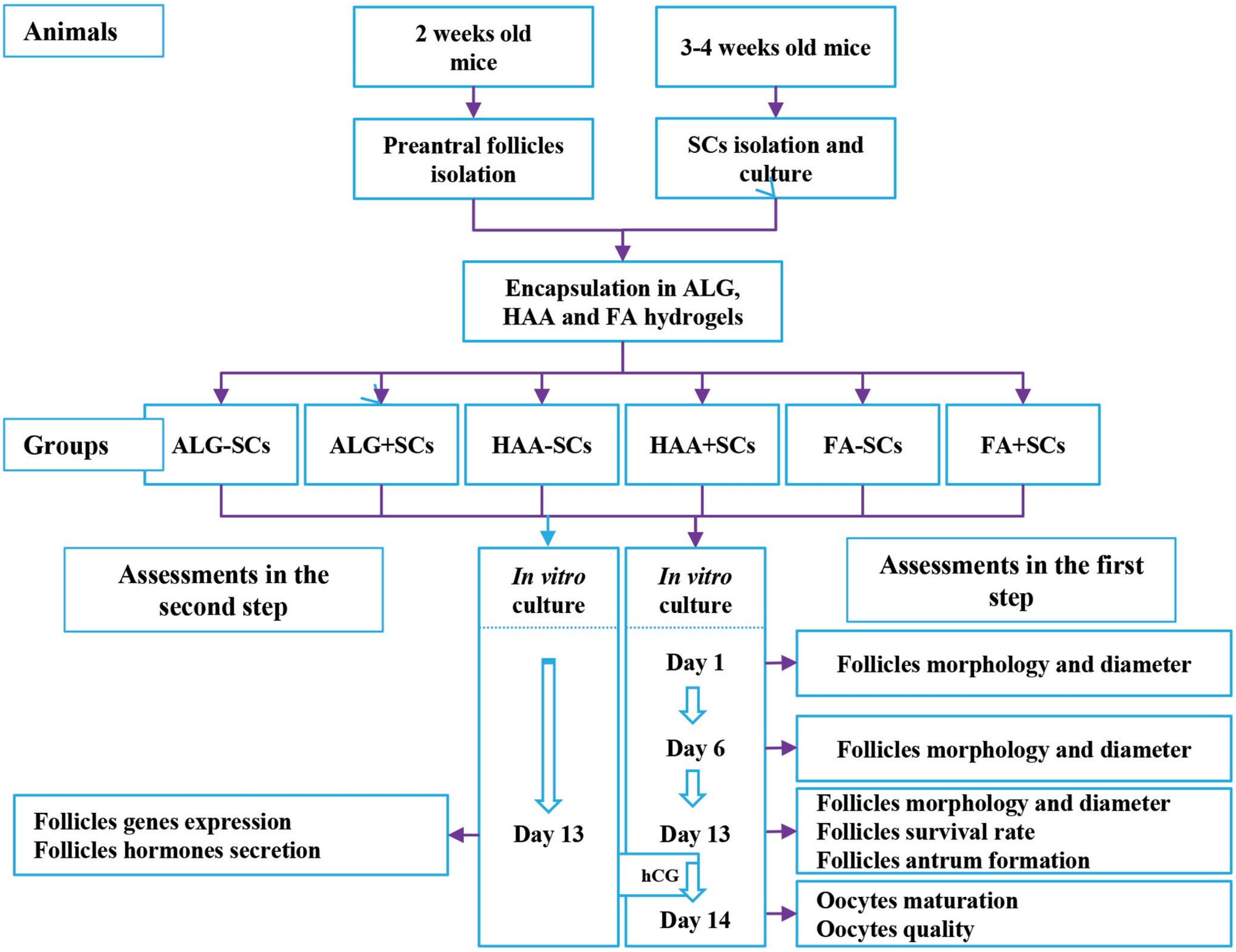

Fig.S1: Graphic representation of the study design.
ALG; Alginate hydrogel, HAA; Hyaluronic acid-alginate hydrogel, FA; Fibrin-alginate hydrogel, -OCs; Culture in the absence of ovarian cells, +OCs; Culture in the presence of ovarian cells, SCs;…., and hCG; Human chorionic gonadotropin.

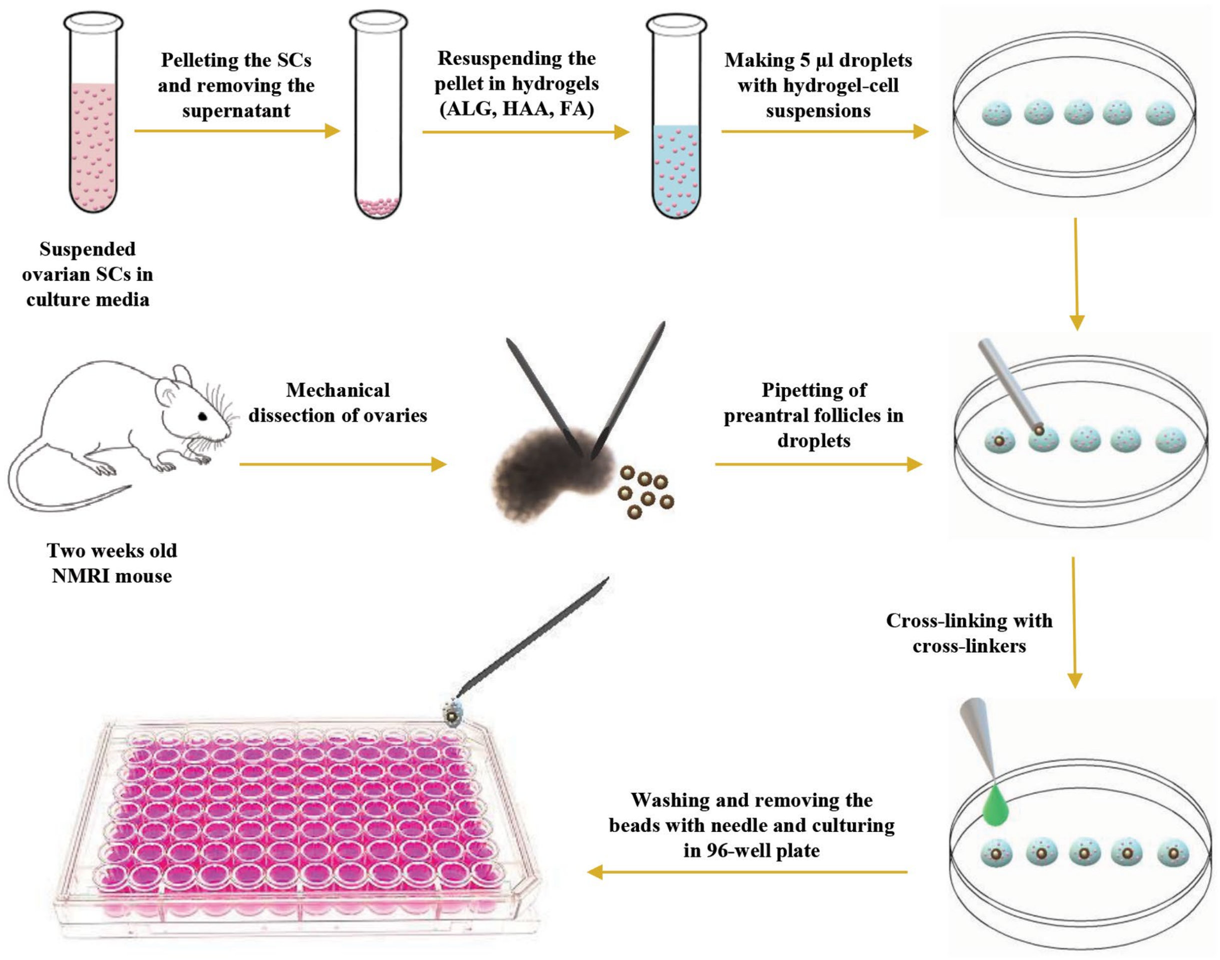

Fig.S2: Flowchart for preantral follicles and OCs co-encapsulated in ALG, HAA and FA hydrogels. OC; Ovarian cells, ALG; Alginate, HAA; Hyaluronic acid-alginate, FA; Fibrin-alginate, and SCs;….

Table S1: Primer sequences used for real-time polymerase chain reaction (PCR) analysis

Gene	Accession number	Primer pair (5′-3′)	Product length (bp)
Gdf9	NM_008110.2	F: CAAACCCAGCAGAAGTCAC<br>R: AAGAGGCAGAGTTGTTCAGAG	194
Bmp15	NM_009757.5	F: AAATGGTGAGGCTGGTAA<br>R: TGAAGTTGATGGCGGTAA	148
Zp3	NM_011776.1	F: CTTGTGGATGGTCTATCTGAG<br>R: GTGATGTAGAGCGTATTTCTG	125
Gja4 (Cx37)	NM_008120.3	F: CGACGAGCAGTCGGATTT<br>R: AGATGACATGGCCCAGGTAG	155
Gja1 (Cx43)	NM_010288.3	F: TAAGTGAAAGAGAGGTGCCCAGA<br>R: GGTTGTTGAGTGTTACAGCGAAAAG	200
Bmp4	NM_007554.3	F: GGTCGTTTTATTATGCCAAGTCC<br>R: ATGCTGCTGAGGTTGAAGAGG	417
Bmp7	NM_007557.3	F: CTATGCTGCCTACTACTGTGAG<br>R: GTTGATGAAGTGAACCAGTGTC	103
Trp53 (P53)	NM_011640.3	F: AACTTACCAGGGCAACTATG<br>R: TGTGCTGTGACTTCTTGTAG	203
Casp3	NM_001284409.1	F: AAAGACCATACATGGGAGC<br>R: CGAGATGACATTCCAGTGCT	138
Bax	NM_007527.3	F: TTGCTACAGGGTTTCATCCAG<br>R: CCAGTTGAAGTTGCCATCAG	246
Bcl2	NM_009741.5	F: GCCTTCTTTGAGTTCGGT<br>R: ATATAGTTCCACAAAGGCATCC	162
Fshr	NM_013523.3	F: ACGCCATTGAACTGAGATTTG<br>R: GAACACATCTGCCTCTATTACC	134
Lhcgr	NM_013582.3	F: AAGCACAGTTAGAGAAGCGA<br>R: GGTCAGGAGAACAAAGAGGA	244
Cyp11a1	NM_019779.4	F: TCCTTTGAGTCCATCAGCAG<br>R: GTCCTTCCAGGTCTTAGTTCT	180
Cyp17a1	NM_007809.3	F: AGAAGTGCTCGTGAAGAAGG<br>R: TTGGCTTCCTGACATATCATCT	201
Cyp19a1	NM_007810.4	F: ATGTCGGTCACTCTGTACTTC<br>R: TTTATGTCTCTGTCACCCACAAC	107
GAPDH	NM_001289726.1	F: GACTTCAACAGCAACTCCCAC<br>R: TCCACCACCCTGTTGCTGTA	125
